# Painful Anal Ulcers in an HIV-Negative Young Woman: An Atypical Presentation of Syphilis

**DOI:** 10.7759/cureus.50575

**Published:** 2023-12-15

**Authors:** Jenna Kroeker, Jose Lopez, Diego Castellon, Yu Shia Lin, Rebecca Rhee, Meredith Pittman

**Affiliations:** 1 Department of Surgery, Maimonides Medical Center, Brooklyn, USA; 2 Department of Surgery, Flushing Hospital Medical Center, Queens, USA; 3 Infectious Diseases, Department of Medicine, Maimonides Medical Center, Brooklyn, USA; 4 Department of Pathology, Maimonides Medical Center, Brooklyn, USA

**Keywords:** histology and histopathology, sexually transmitted infection (sti), treponema pallidum, perianal lesion, anorectal disease, anal syphilis

## Abstract

Anorectal syphilis is relatively uncommon and diagnostically challenging given the wide differential diagnosis for anal lesions. Risk factors, such as men who have sex with men or HIV-positive status, are especially important to elicit from patients during the clinical history. In this report, we present a rare case of painful anal syphilis diagnosed in an HIV-negative woman by tissue biopsy*. *

## Introduction

The incidence of syphilis, a sexually transmitted infection (STI) caused by the spirochete bacterium *Treponema pallidum*, continues to rise in the United States at an alarming rate. In the year 2000, the reported rate of infection was 11.2 per 100,000; in 2021, it was 53.2 per 100,000. [1, 2]. Most of these cases are found in men who have sex with men (MSM), patients who have human immunodeficiency virus (HIV), or patients who have other "high-risk" behaviors, such as intravenous drug use [1]. The classic lesion of primary syphilis is a solitary, painless genital ulcer or chancre. The presentation of secondary syphilis can be more variable with a fever, rash, and/or lymphadenopathy. A wide variety of less common disease manifestations are possible, earning syphilis the epithet of "the great imitator" [2].

Despite a rise in the reported cases of syphilis, infections secondary to *Neisseria gonorrhea* and *Chlamydia trachomatis* remain the more common, and therefore the more commonly tested for, STIs [3]. Additionally, traditional screening with nontreponemal serologic tests is known to give false negative results in the early stages of disease [4]. For these reasons, syphilis may go unrecognized by clinicians in non-classical settings.

In this report, we present a rare case of anal syphilis in a young woman who presented with perianal pain. A comprehensive literature search yielded no reported cases of anal syphilis in a woman in the United States.

## Case presentation

A 23-year-old otherwise healthy woman was referred to the outpatient colorectal surgery clinic for evaluation of a painful perianal lesion that had been present for two months. The patient’s pain was localized to the anal verge and exacerbated during defecation. The patient described clear drainage coming from the lesion without associated dysuria, hematuria, rash, or ulcers in her oral cavity or genitalia. Her history was notable for sexual intercourse with multiple partners and inconsistent barrier protection. However, STI testing was unremarkable one year prior to presentation, including a treponemal antibody screening test. Repeat serologic studies were performed and were negative, as were nucleic acid amplification testing (NAAT) for* Neisseria gonorrhea* and *Chlamydia trachomatis*. She had recently completed a 3-day course of oral Bactrim antibiotics prescribed by her gynecologist but she had not experienced symptomatic improvement.

On physical exam, bilateral perianal ulcerations were identified. These were tender to palpation and weeping clear fluid. Herpes simplex virus (HSV) was presumed based on history and examination and the patient was treated with two courses of valacyclovir, again without improvement of her symptoms.

**Figure 1:**
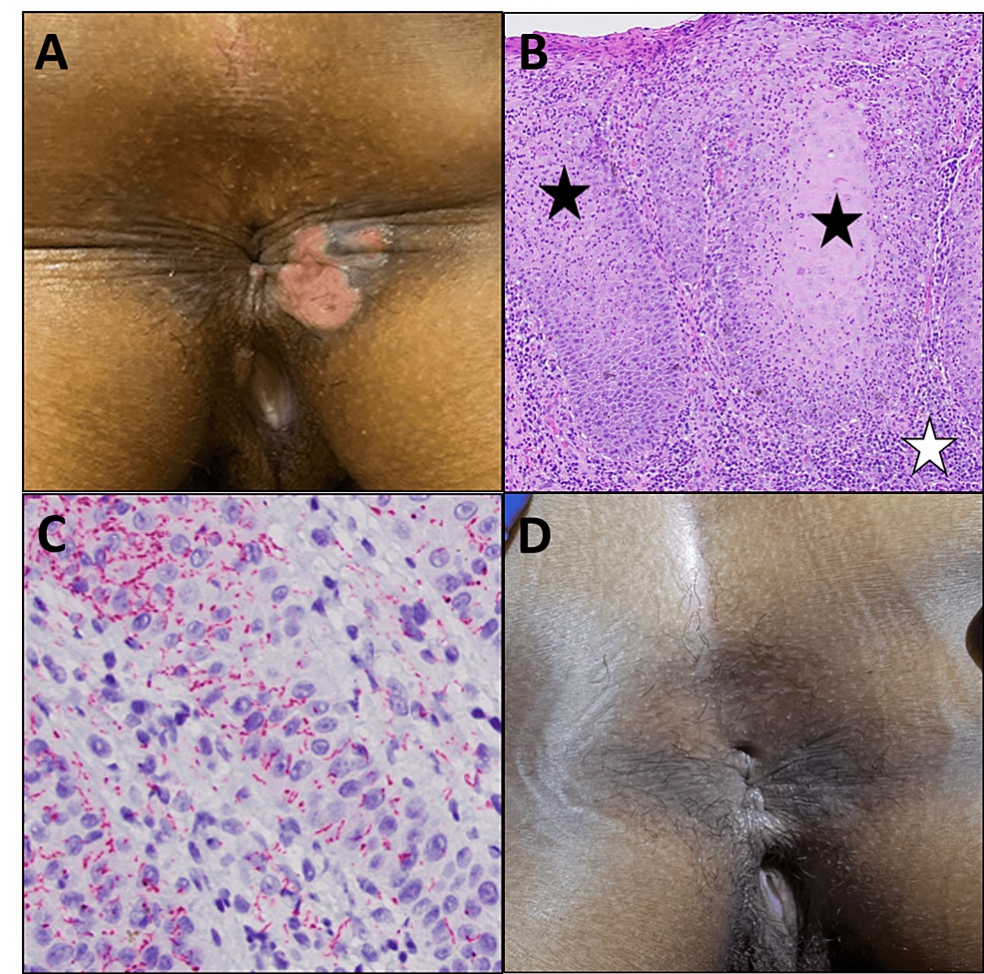
Syphilitic perianal ulcers A, Shallow perianal ulcers with sharp borders were identified. These lesions were painful to touch and weeping clear fluid. B, Histopathologic review of a tissue biopsy showed inflamed squamous mucosa (black star) with dense, plasma cell-rich inflammation (white star) and atypical histiocytes with clear cytoplasm (hematoxylin and eosin stain, 200x). C, The *Treponemal* organisms in the mucosa were highlighted by a spirochete stain (red chromagen, 400x). D, After treatment with Penicillin G, the patient experienced complete resolution of her symptoms and the anal lesion.

The patient returned to the clinic for a rectal exam under anesthesia and a biopsy of the perianal lesion. This examination showed a shallow ulcer at the anal verge with a sharp, raised border (Figure 1A).

The ulcer was tender but did not have purulent discharge. A biopsy from the ulcer edge was sent to pathology and showed ulcerated squamous mucosa with abundant plasma cell inflammation and atypical appearing histiocytes with cleared cytoplasm. (Figure 1B). A syphilitic ulcer was suspected due to the clinical history and histologic features, and a spirochete immunostain confirmed *Treponemal pallidum* infection (Figure 1C).

The patient was referred to an Infectious Disease specialist for further workup and management. A comprehensive serologic panel was sent and showed that the patient was *Treponema* antibody and *Treponema pallidum* particle agglutination assay (TPPA) positive, but rapid plasma reagin (RPR) and HIV negative. She was treated with one dose of intramuscular penicillin. Her perianal lesion and associated pain resolved entirely on examination (Figure 1D).

## Discussion

The incidence of sexually transmitted infections in the United States has steadily increased over the past decade [3]. Although primary and secondary (P&S) syphilitic infections reached an all-time reported low in the year 2000, by 2017, cases had increased by >400%, and this rising trend continued into the COVID-19 pandemic [1]. In general, men make up between 80-90% of newly reported P&S syphilis cases, and most of these patients identify as men who have sex with men (MSM) [1]. Still, the rate of P&S syphilis in women has also been increasing, and the concerning rise in incidence across multiple populations means that syphilis should remain on the differential diagnosis for all patients who present with uncharacteristic anal lesions [1, 2].

The differential diagnosis for an anorectal lesion is broad. A painful perianal ulcer or fissure in a midline position is most often related to trauma/constipation [5]. If there are multiple fissures, or if the fissures are in a lateral position, then inflammatory bowel disease, specifically Crohn's disease, should be considered [5,6]. If an ulcer or mass is present, then neoplasia may be favored, and a biopsy is required for appropriate diagnosis. Ceretti et al reported a patient who identified as an MSM and who presented with a 3 cm ulcerated right rectal mass that was palpable on digital rectal examination and highly concerning for malignancy. A tissue biopsy confirmed primary syphilis rather than carcinoma [7]. Similar mass-like presentations causing clinical concern for neoplasia have been reported in other anatomic sites, including the stomach and oral cavity [8-9]. Finally, patients who present with the classic painless genital chancre of primary syphilis may receive testing for STIs; however, up to one-third of perianal syphilitic lesions may be painful. The presence of pain is not known to correlate with co-infection with either HSV or HIV [10].

Given this wide differential for perianal lesions and the variability in presentation for anal syphilis, additional testing is often necessary to reach a diagnosis of anal syphilis. If a tissue biopsy is taken, histologic clues to a diagnosis of syphilis include a plasma cell-rich inflammatory infiltrate, frequent lymphoid aggregates, and a relative lack of neutrophils and eosinophils [11]. In our case, unusual histiocytes with abundant pale cytoplasm were also a distinct morphologic feature that raised suspicion for an infectious etiology.

For serologic diagnosis of syphilis, there are two primary testing algorithms. In the first traditional diagnostic pathway, nontreponemal tests, such as the venereal disease research laboratory (VDRL) assay and RPR test, are performed as screening assays due to their high sensitivity (>85% in primary and secondary syphilis). This algorithm is limited by false negative results that occur early in Treponemal infection, which may have been the case with our patient. [4,12] The reverse screening algorithm begins with a treponemal-specific test, such as a *T. pallidum* particle agglutination test. Although the treponemal tests are more sensitive in early infection, they have occasional false positive results and remain positive indefinitely, negating any ability to monitor patient response to therapy or re-infection [12]. When an STI is suspected in the setting of an anogenital lesion, performing concurrent syphilis serologies and chlamydia/ gonorrhea nucleic acid amplification testing (NAAT) is prudent given the high likelihood of STI co-infection [3]. In the setting of a non-genital or perianal site of the lesion, biopsy can provide important information to rule out other etiologies and confirm the presence of spirochetes [11].

Prompt diagnosis and treatment of syphilis prevents disease progression in the patient and reduces disease transmission within a population. Penicillin is a highly effective treatment for P&S syphilis; there are no documented cases of penicillin resistance in over 60 years of use for the treatment of *Treponema pallidum* [2]. A single dose of 2.4 million units of benzathine penicillin G is the current treatment standard for early syphilis with a low treatment failure rate of 5% [2]. For our patient, the delay in diagnosis of syphilis led to the unnecessary treatment of HSV with valacyclovir and prolonged disease symptoms. Once diagnosed, a single dose of Benzathine penicillin G was effective in resolving the patient’s symptoms and clearing the infection.

## Conclusions

This case documents an atypical, painful presentation of anal syphilis in a young HIV-negative woman whose only risk factor appears to have been multiple sexual partners. Ultimately, the detection and timely treatment of P&S syphilis relies on data from a comprehensive clinical history, appropriate laboratory testing, and occasional histopathologic review. With ever increasing rates of sexually transmitted infections, clinicians can expect to see more patients with routine and atypical presentations of these pathogens. Prompt penicillin G therapy is effective and can prevent patient morbidity and mortality from advanced syphilitic disease.
